# The Influence of Intensive Nutritional Education on the Iron Status in Infants

**DOI:** 10.3390/nu14122453

**Published:** 2022-06-14

**Authors:** Dagmara Woźniak, Tomasz Podgórski, Patrycja Krzyżanowska-Jankowska, Małgorzata Dobrzyńska, Natalia Wichłacz-Trojanowska, Juliusz Przysławski, Sławomira Drzymała-Czyż

**Affiliations:** 1 Department of Bromatology, Faculty of Pharmacy, Poznan University of Medical Sciences, Rokietnicka 3 Street, 60-806 Poznań, Poland; dagmara.wozniak@student.ump.edu.pl (D.W.); mdobrzynska@ump.edu.pl (M.D.); jotespe@ump.edu.pl (J.P.); 2 Faculty of Pharmacy, Doctoral School, Poznan University of Medical Sciences, Fredry 10 Street, 61-701 Poznań, Poland; 3 Department of Physiology and Biochemistry, Poznan University of Physical Education, Królowej Jadwigi 27/39 Street, 61-871 Poznań, Poland; podgorski@awf.poznan.pl; 4 Department of Pediatric Gastroenterology and Metabolic Diseases, Faculty of Medicine, Institute of Pediatry, Poznan University of Medical Sciences, Szpitalna 27/33 Street, 60-572 Poznań, Poland; pkrzyzanowska@ump.edu.pl; 5 Healthcare Center, Partyzantów 14 Street, 64-510 Wronki, Poland; wichlacz.natalia@gmail.com

**Keywords:** child nutrition, development, early nutrition, nutritional programming, transferrin, ferritin

## Abstract

Iron is an essential nutrient for a child’s proper development at every growth stage. It is crucial for the production of red blood and muscle cells, DNA replication, and the development of the brain, nervous and immune systems. Iron deficiency is the most common micronutrient deficiency in children worldwide. Despite widespread access to nutritional information for children, parents continue to make many feeding mistakes. This study aimed to assess whether any nutritional intervention would affect the iron status in children. The parents of 203 children were randomly assigned to one of two groups: the study group received intensive mobile nutritional education for a year, while the control group received no intervention. Blood tests were performed on both groups at the beginning of the study and one year later. The educational intervention resulted in statistically significantly higher levels of RBC (red blood cells; *p* = 0.020), HGB (haemoglobin; *p* = 0.039), HCT (haematocrit; *p* = 0.036), MCV (mean cell volume; *p* = 0.018) parameters and iron dietary intake (*p* ≤ 0.001). Even a non-targeted dietary intervention improves the iron status in children. As iron management is insufficient in most children, an iron-targeted nutritional intervention appears necessary.

## 1. Introduction

Iron is an essential nutrient for a child’s proper development at every growth stage [1]. Iron is crucial for the production of new red blood and muscle cells, DNA replication, and the development of the brain, nervous and immune systems [1,2,3]. Iron deficiency in infants can result in poor memory and attention, a higher risk of attention-deficit hyperactivity disorder, visual and auditory system impairment and social and emotional behaviours [1,4,5,6,7,8,9]. Iron is mainly found in haemoglobin in RBC, erythroblasts, myoglobin in muscles, and other proteins such as transferrin or ferritin [1,10,11]. Iron deficiency is the most common micronutrient deficiency in children worldwide [1]. According to research, in 2010, global anaemia prevalence was 32.9%, with iron deficiency being the leading cause [12,13]. It is estimated that in Europe, iron deficiency affects 4–18% of children aged 6 to 12 months [14]. Iron deficiency and its result—iron deficiency anaemia, can affect growth and energy levels and impair motor and cognitive development in children [1,15]. Although iron supplementation can treat anaemia, there is limited evidence that it can restore neurodevelopmental impairments [1,16,17]. In the early stages of life, iron requirements are high. Therefore, for proper development during the first six months after birth, it is crucial for the foetus to acquire adequate iron stores from its mother during the pregnancy. The best dietary source for infants is breast milk. The iron contents of breast milk are extremely low but highly bioavailable [1,18]. Around the sixth month of age, the iron reserves in the baby’s body and mother’s milk are insufficient to meet the growing demands. Therefore, there is a need to expand the child’s diet with solid products [1].

Nutritional programming states that both an excess and a deficiency of nutrients in the first 1000 days of a child can have long-term effects on developing tissues and reprogram the metabolism [19,20,21]. This has a huge impact on health in adulthood [19,20,21]. Nutritional programming clearly demonstrates the correlation between child malnutrition and adult prevalence of cardiovascular disease, obesity, osteoporosis, hypertension, impaired glucose tolerance, insulin resistance and type 2 diabetes [22,23,24,25,26]. Nutritional programming is also important for ensuring an adequate supply of micronutrients [19,20,21]. Despite widespread access to nutritional information for children, parents continue to make numerous feeding mistakes. According to the PITNUTS study, parents expanded their babies’ diet too soon and gave their children too many meals, especially snacks [27]. As a result, children’s diets were excessively high in energy, simple sugars and protein, while low in long-chain polyunsaturated fatty acids (LCPUFA), vitamin D, potassium, fats, vitamin E, calcium and fibre [27]. The diet was, therefore, deficient in both macro-and micronutrients. Proper nutrition for children cannot be ensured unless parents have an adequate level of nutritional knowledge.

Thus, this study’s goal was to assess whether any nutritional intervention would affect iron status in children.

## 2. Materials and Methods

### 2.1. Participants

The study was conducted on a group of parents of 203 Polish infants. Parents were randomly assigned to one of two subgroups. Parents of 102 infants received intensive mobile nutritional education for around a year. Parents of the other 101 infants were served as the control group. The planned intervention involved intensive nutritional education delivered to parents through short text messages about their children’s nutrition (approximately 4–6 times a week). The content of text messages was personalised to a few conditions such as infant age, stage of development or season of the year. A certified dietitian developed a nutritional education plan based on official infants’ nutrition guidelines [28,29]. The messages described the positive influence of breastfeeding on children’s health. Furthermore, the messages had tips on the introduction of solid food to the infants’ diets (taking into account the selection of the right time, type of food and portion), as well as the principles of responsive feeding (appropriate response to hunger and satiety cues from babies). Infants between the ages of 6 and 8 weeks were mainly recruited for the study during the first vaccination schedule. The study was conducted in five Paediatric Outpatient Clinics in Poland during 2019–2022.

Parents of 160 children completed the study. Parents of nine children were lost due to follow-up, parents of 13 children failed to appropriately complete the nutrition diary for their children, and 21 children were given iron during the study—thus, their blood parameters were not included in the statistical analyses. Both at the beginning of the study and after the intervention, the parameters of iron status in children were examined.

The majority of respondents lived in the village from the city agglomeration (60%) and held a university degree (76%). Table 1 presents the characteristics of the groups.

Inclusion criteria were children born between the 36th and 42nd week of pregnancy, children under the age of eight weeks and written consent from children’s parent or legal guardian. In addition, each child received a minimum Apgar score of eight at birth.

Exclusion criteria include children with birth weights below 2500 g, a history of chronic systemic disease, gastrointestinal diseases resulting in digestion and absorption disorders, and other severe systemic diseases (cancer, endocrinopathies, connective tissue diseases, kidney diseases and diabetes).

### 2.2. Statistical Analyses

Power analyses were carried out using G*Power v. 3 (University of Dusseldorf, Dusseldorf, Germany). Assuming the 80% power of the test and the significance level of 5% (two-tailed), the study necessitated data from 158 patients. The predicted percentage of patients lost to follow-up was 20%, yielding the final group size of 198 children. Therefore, 203 children were recruited and randomized.

Obtained data were analysed using MedCalc 19.6 (MedCalc Software, 1993–2020) and GraphPadPrism 5.01 (GraphPad Software, Inc., La Jolla, CA, USA) statistical software. For all parameters, medians and 1st–3rd quartiles were calculated unless indicated otherwise. The Shapiro–Wilk test was used to check the normality of the data distribution. Statistical differences between groups were tested using Mann–Whitney and Fisher’s tests. A *p*-value of <0.05 was considered statistically significant. Statistical analyses were conducted on the basis of descriptive statistics. The nutritional status of the children was evaluated based on the standardized Z-score for weight concerning the cut-off points established by the WHO [30]. The standard weight falls within the range of the Z-score from −2 SD to 1 SD, underweight: <−2 SD to −3 SD, overweight: 1 SD to <2 SD, obese from 2 SD to 3 SD.

### 2.3. Ethical Consideration

All subjects gave their informed consent for inclusion before participating in the study. Parents were asked to read the informed consent form and consent to collect and process the personal data form. Then, they were asked to consent to their participation and the data processing. The research was conducted in accordance with the Declaration of Helsinki, and the Bioethical Committee approved the protocol of the Poznan University of Medical Sciences in Poznań, Poland (decision no 723/19).

### 2.4. Blood Collection

The blood was collected by qualified, professional laboratory personnel. All safety precautions were strictly followed during blood collection. At the beginning of the study, blood samples were obtained from a heel prick using a disposable Medlance^®^ Red lancet-spike (HTL-Zone, Berlin, Germany) with a 1.5 mm blade and 2.0 mm penetration depth. At the end of the study, the same equipment was used to collect the blood from the heel prick or finger (depending on the possibility). We decided to collect capillary blood to minimise children’s suffering and fear. This method allowed for the collection of small amounts of blood for analysis. A total of 300 μL of capillary blood was collected in a Microvette^®^CB 300 Z tube (Sarstedt, Nümbrect, Germany) with a clotting activator for biochemical measurements. The blood was centrifuged (1500 g, 4 °C, 10 min) for serum separation. Additionally, 300 μL of capillary blood was collected into a Microvette^®^ CB 300 tube (Sarstedt, Nümbrect, Germany) containing K2-EDTA (EDTA dipotassium salt) as anticoagulant for haematological measurement.

### 2.5. Hematological and Biochemical Measurements

Blood sample tests were carried out with the use of an 18-parametric automated haematology analyser Mythic^®^ 18 (Orphée, Geneva, Switzerland). The reader aspirated only 9.8 μL of blood during the measurement. The red blood cell (RBC) count, haemoglobin (HGB) concentration and haematocrit (HTC) value were considered in the study.

Within freshly separated serum, the concentration of iron (ACCENT-200 FERRUM, Cormay, Cat No. 7-258, Łomianki, Poland) was measured using a colorimetric method. Furthermore, transferrin (ACCENT-200 TRANSFERRIN, Cormay, Cat No. 7-210, Łomianki, Poland) and ferritin (ACCENT-200 FERRITIN, Cormay, Cat No. 7-230, Łomianki, Poland) concentrations were measured as a result of antigen–antibody reactions with the specific antibodies. All biochemical parameters were determined using the Accent 220S (Cormay, Łomianki, Poland) biochemical analyser. The sensitivity of the methods for serum iron, transferrin and ferritin were 4.1 µg/dL, 0.076 g/L and 9.1 ng/mL, respectively. The coefficients of variation (CVs) for the iron assay were 1.10% and 1.87%, transferrin were 1.12% and 4.56% and ferritin were 1.5% and 4.0%, respectively to the repeatability- and reproducibility-assay CVs.

### 2.6. Dietary Intake

Parents provided information about their children’s menu over a 3-day period on average, including meals, snacks and fluids. This eating schedule had to include one weekend day. Before completing the last part, parents were trained by a dietitian and instructed on how to complete the food diary. The instruction contained simple hints on filling in the food diary correctly and a link to the page to help determine the proper food portions (www.ilewazy.pl, accessed on 11 August 2009). Forms of contact (e-mail and telephone number) to the dietitian were also provided in case of questions.

Children’s diets were analysed using Dietetyk 2015 (Jumar Software, Poznan, Poland). The NutritionData.com database was also applied in the study. The average daily intake of micro- and macronutrients was calculated for children from each group and compared with the recommended dietary allowance (RDA) according to Polish nutritional standards [31]. We analysed the supply of nutrients in children’s diets to evaluate their influence on iron status in children. When analysing each meal, technological losses (e.g., cooking, frying) were considered. The dietary supplementation was not included in the assessment.

## 3. Results

Table 2 presents birth weight in children.

Both groups underwent blood tests at the beginning of the study and one year later. At the baseline, parameters in both groups did not differ statistically significantly (Table 3).

The comparison of the initial and final results of blood tests revealed that the study group showed statistically significantly higher levels of RBC, HGB, HCT and MCV parameters (Table 4). The following table demonstrates that the iron parameter changed in the study group, but that the changes occurred in indirect parameters rather than direct parameters.

Comparing the delta values of blood parameters showed statistically significant lower ferritin losses in the study group (Table 5).

We also analysed whether the measurements fell within the reference ranges. The control group was characterised by a number of features of iron deficiency anaemia, including lower RBC, HGB, HCT and MCV, along with elevated transferrin (Table 6).

Finally, the amount of iron consumption and selected macro-and micronutrients that may affect iron absorption, such as protein, fibre and vitamin C, were estimated. After one year of dietary education, children in the study group had statistically higher dietary intakes of iron, fibre and vitamin C (Table 7). In addition, their diets were also more varied than those of their peers in the control group, containing significant amounts of vegetables, fruits, various sources of complex carbohydrates (groats, pasta, rice) and complete protein in the form of lean meat and dairy.

## 4. Discussion

As previously stated, the aim of the study was to assess the impact of intensive nutritional education, carried out by sending text messages to the parents’ mobile devices, on the iron status of the infants in the study. It should be emphasised that this is the first study involving this type of education addressed to the parents of youngest children, and the messages sent contained information on general principles of infant nutrition, diet expansion, and the proper reading of the signs of hunger and satiety. It is the first study to assume that nutritional education should begin at a young age.

Various factors can influence the iron status in children, such as socioeconomic status, demography, the type of milk consumed by infants, dietary intake, elevated inflammation levels (e.g., C-reactive protein) and gastrointestinal diseases [15,18,34,35,36,37,38]. Different studies confirmed that children from families from lower socioeconomic backgrounds, schooling and employment status; rural areas; and consuming less meat, fruits, and more cow milk are more prone to iron deficiency [34,35,36,37,38]. Thus, outside of our parental nutritional education, many factors can influence iron status during infancy.

According to research, iron deficiency is common in infants’ diet [15]. Dietary iron can be heme or non-heme. Compared with non-heme iron, heme iron has higher bioavailability [2]. Heme iron can be found in animal proteins such as fish, poultry and meat, while non-heme iron can be found in vegetable sources [15]. Therefore, the adequate introduction of complementary food is necessary to ensure sufficient iron supply in the diet of children. In this research, mothers learnt how to expand their children’s diets properly. They received detailed guidelines on the correct time of introducing complementary meals, the sequence of introducing new products, and ideas for meals. Important information for mothers was the appropriate amount of nutrition that the baby should receive and the frequency of serving meals. The acquired knowledge was implemented in the diets of children. Children in the study group consumed more vegetables and fruits than children in the control group—their diets were more varied. There were also animal and plant sources of iron. Greater diversity and the courage of mothers in introducing new meals to their children significantly increased the supply of fibre, vitamin C and iron in the diets of the children in the study group. The nutrition of the control group consisted mainly of products intended for children, including a large supply of modified milk and porridges for infants.

The positive effects of nutritional education on iron status in children have already been shown in different studies. There has been limited research in the world literature on the influence of nutritional education on the iron status in children that can be related to the European population. Most of the interventions were undertaken in third world or low-income countries [39,40,41,42]; these are countries with populations that are culturally and nutritionally different from European populations. Moreover, most nutritional education studies involved only children with iron deficiency or anaemia [43,44,45]. There have been no studies that show the effectiveness of interventions on such a young age group; however, the following studies can be relevant to our findings. Choi et al. showed that children with more educated mothers were less likely to develop anaemia (*p* = 0.032) and iron deficiency (*p* = 0.058) than were those with less-educated mothers [46]. Children with more educated mothers consumed more protein (*p* < 0.001) and iron (*p* = 0.001) from animal sources. There was a significant inverse relationship between maternal education and the prevalence of anaemia (odds ratio: 0.52; 95% confidence interval: 0.32, 0.85) [46]. In our study, the iron parameter does not differ between the groups. However, iron concentration is strongly dependent on inflammation, infection and the overall state of health; therefore, it is not the best parameter to assess iron status.

Khoshnevisan et al. carried out a three-month nutritional education programme for mothers of pre-school children. They found that ferritin concentrations differed significantly between groups, with a reduction in the control and an elevation in the nutrition education groups [47]. In our study, the final concentration of ferritin does not differ significantly. The change in the concentration of ferritin between the beginning and the first year of life is enormous and in the study group, it was less rapid. However, it should be noted that huge changes in serum ferritin from the study-beginning to study-end are as to be expected when moving from a young infant into early childhood [48,49]. The newborn ferritin values decrease until the end of the first year and stabilize thereafter in a sex-specific fashion [49].

Al-Suhiemat et al. discovered a statistically significant relationship between maternal educational level and haemoglobin level (χ^2^ = 8.820, *p* = 0.012) [50]. Change in the haemoglobin level was also confirmed in our study. The concentration of haemoglobin was below the reference values in 25% of patients in the study group. However, it is still better compared to the control group.

However, our study is the first to start education for children at this early age and engage both parents in a very simple and low-cost way using modern technology. Furthermore, it included a very specific group of patients in terms of nutrition of which there are many limitations, and on the other hand, the obtained effect may permanently programme the child’s metabolism.

Determination of blood parameters in all children were performed on capillary blood. Some doubts may arise from the fact that the capillary blood samples have a higher haemoglobin concentration than venous blood samples. However, it should be emphasized that all of the reference values used were created for the parameters determined in capillary blood and they took into account the patient’s age. It was up to the paediatrician to decide whether the obtained blood results indicating deficiencies in haemoglobin and/or other parameters levels should be referred for further diagnosis or treatment.

Maintaining patients in a trial that includes one year of intervention and two follow-up measures (at the start and end of the trial) may be challenging. To maintain the interest of patients, text messages regarding nutritional education were individually matched to the age, health and progress of the growing child’s diet. Moreover, the mother in the study group had the opportunity to contact a dietitian at any time to clarify her nutrition concerns. Patients in the control group did not receive nutritional education. Following the random assignment to groups, some control patients dropped out of the study, resulting in an extended recruitment period.

The parents were always concerned about correctly filling out their nutritional diaries. However, the parents were trained by a dietitian before filling out the diaries, and most of them were also college educated.

There may also be concerns that only the parents are responsible for feeding their children. Commonly, the closest members of the rest of the family also influence the child’s nutrition. Nevertheless, in an interview with a dietitian, parents indicated who was responsible for their children’s nutrition, and in 85% of cases, it was only the mother, 5% the father, and 10% for both parents.

The limitation of the study is that it only examines the short-term effects of education. It would be worthwhile to re-check the groups after a few years (e.g., 5, 10 years) to see how the parents’ education in early childhood and the improvement of iron parameters influenced the children’s later health. Especially, as the child gets older, the circle of people who have an impact on their nutrition (e.g., peers) grows. Furthermore, the impact education has on cognitive functions in infants has not been checked.

While oral ferrous sulphate is the cheapest and most effective therapy for iron deficiency anaemia, simple nonpharmacological measures such as nutritional education can help immensely in the prevention of iron deficiency [15,51,52].

## 5. Conclusions

Even a non-targeted dietary intervention improves the iron status in children (dietary iron intake, RBC, HGB, HCT, MCV). In addition, education causes a smaller drop in ferritin concentration during the first year of an infant’s life in the study group. However, the reduced haemoglobin concentration occurs even if nutritional education is carried out. Nutritional education does not completely protect against the occurrence of decreased haemoglobin concentration, but it definitely influences the improvement of iron status. As iron management is insufficient in most children, it appears that an iron-targeted nutritional intervention is required. Thus, it is crucial to provide the nutritional programme for parents.

## Figures and Tables

**Table 1:** Data concerning parents’ age, education and place of residence.

Parameters	Parents GR 1	Parents GR 2	*p*
	Median (1st–3rd Quartile)		
Age (years) ^1^	30 (28–34)	30 (28–34.5)	ns
Place of residence ^1^			ns
Village (from the city agglomeration)	63%	58%	
A city with fewer than 500,000 residents	26%	30%	
A city with more than 500,000 residents	11%	12%	
Education ^1^			ns
Primary	0%	3%	
Secondary	25%	20%	
Higher	75%	77%	

GR 1—study group; GR 2—control group; ^1^ Mann–Whitney test; ns—not significant.

**Table 2:** Body weight in children.

Parameters	GR 1 (*n* = 80)	GR 2 (*n* = 80)	*p*
	Median (1st–3rd Quartile)		
Birth weight (g) ^1^	3500 (3300–3710)	3598 (3170–3835)	ns
Z-score for birth weight ^1^	0.686 (0.294–1.098)	0.878 (0.039–1.343)	ns

GR 1—study group; GR 2—control group; ^1^ Mann–Whitney test; ns—not significant.

**Table 3:** Comparison of anaemia-related parameters between the study groups at the baseline.

Parameters	GR 1 (*n* = 80)	GR 2 (*n* = 80)	*p*
	Median (1st–3rd Quartile)		
RBC (10^12^/L) ^1^	3.845 (3.420–4.210)	3.930 (3.260–4.460)	ns
HGB (mmol/L) ^1^	6.770 (6.580–7.950)	6.960 (6.460–7.760)	ns
HCT (L/L) ^1^	0.371 (0.303–0.361)	0.307 (0.286–0.362)	ns
MCV (fL) ^1^	83.700 (79.900–87.900)	82.400 (77.600–90.400)	ns
MCH (fmol) ^1^	1.890 (1.710–1.930)	1.880 (1.660–2.040)	ns
MCHC (mmol/L) ^1^	21.920 (21.180–22.230)	22.420 (20.870–22.980)	ns
Iron (µg/dL) ^1^	70.100 (53.600–79.700)	74.500 (56.600–87.400)	ns
Transferrin (g/L) ^1^	1.990 (1.860–2.460)	2.300 (1.956–2.720)	ns
Ferritin (ng/mL) ^1^	171.100 (76.300–308.100)	134.800 (37.200–227.700)	ns

GR 1—study group; GR 2—control group; ^1^ Mann–Whitney test; RBC—red blood cells; HGB—haemoglobin; HCT—haematocrit; MCV—mean cell volume; MCH—mean corpuscular haemoglobin; MCHC—mean cell haemoglobin concentration; ns—not significant.

**Table 4:** Comparison of anaemia-related parameters between the study groups at the end of the trial-end versus end.

Parameters	GR 1 (*n* = 80)	GR 2 (*n* = 80)	*p*
	Median (1st–3rd Quartile)		
RBC (10^12^/L) ^1^	4.300 (4.000–4.500)	4.100 (3.700–4.500)	0.020
HGB (mmol/L) ^1^	7.000 (6.525–7.400)	6.600 (6.100–7.300)	0.039
HCT (L/L) ^1^	0.329 (0.317–0.342)	0.311 (0.287–0.349)	0.036
MCV (fL) ^1^	74.800 (72.600–78.700)	76.100 (74.950–79.500)	0.018
MCH (fmol) ^1^	1.560 (1.530–1.680)	1.640 (1.545–1.680)	ns
MCHC (mmol/L) ^1^	21.390 (20.590–21.740)	21.360 (20.890–21.610)	ns
Iron (µg/dL) ^1^	63.800 (49.500–94.000)	69.900 (41.500–89.900)	ns
Transferrin (g/L) ^1^	3.500 (3.200–3.790)	3.475 (3.100–4.410)	ns
Ferritin (ng/mL) ^1^	16.800 (11.300–28.500)	19.500 (12.200–25.700)	ns

GR 1—study group; GR 2—control group; ^1^ Mann–Whitney test; RBC—red blood cells; HGB—haemoglobin; HCT—haematocrit; MCV—mean cell volume; MCH—mean corpuscular haemoglobin; MCHC—mean cell haemoglobin concentration; ns—not significant.

**Table 5:** Comparison of anaemia-related parameters between the study groups at the end of the trial-delta versus delta.

Parameters	GR 1 (*n* = 80)	GR 2 (*n* = 80)	*p*
	Median (1st–3rd Quartile)		
ΔRBC (10^12^/L) ^1^	0.600 (−0.400–0.900)	0.200 (−0.300–0.375)	ns
ΔHGB (mmol/L) ^1^	0.200 (−1.600–0.600)	−0.300 (−1.200–0.250)	ns
ΔHCT (L/L) ^1^	0.020 (−0.051–0.045)	−0.021 (−0.041–0.017)	ns
ΔMCV (fL) ^1^	−5.400 (−13.600–−2.300)	−6.600 (−10.280–−3.300)	ns
ΔMCH (fmol) ^1^	−0.200 (−0.400–−0.100)	−0.200 (−0.300–−0.100)	ns
ΔMCHC (mmol/L) ^1^	−1.200 (−1.925–0.010)	−0.800 (−1.200–−0.100)	ns
Δiron (µg/dL) ^1^	1.800 (−19.400–18.300)	1.300 (−37.200–14.300)	ns
Δtransferrin (g/L) ^1^	1.200 (0.700–2.000)	1.500 (1.100–2.100)	ns
Δferritin (ng/mL) ^1^	−144.900 (−291.200–−58.200)	−61.000 (−204.300–−15.400)	0.009

GR 1—study group; GR 2—control group; Δ – delta – the difference between the end and the beginning of the study value parameter; ^1^ Mann–Whitney test; RBC—red blood cells; HGB—haemoglobin; HCT—haematocrit; MCV—mean cell volume; MCH—mean corpuscular haemoglobin; MCHC–mean cell haemoglobin concentration; ns—not significant.

**Table 6:** Comparison of anaemia-related parameters between the study groups at the end of the trial.

Parameters	GR 1 (*n* = 80)		GR 2 (*n* = 80)		*p*
	Low	Norm	Low	Norm	
RBC (10^12^/L) ^1^	0	80	8	72	0.007
HGB (mmol/L) ^1^	20	60	38	42	0.005
HCT (L/L) ^1^	0	80	8	72	0.002
MCV (fL) ^1^	0	80	12	68	<0.001
MCH (fmol) ^1^	5	75	4	76	ns
MCHC (mmol/L) ^1^	0	80	0	80	ns
Iron (µg/dL) ^1^	13	67	22	58	ns
Transferrin (g/L) ^1^	17	63	32	48	0.016
Ferritin (ng/mL) ^1^	10	70	5	75	ns

GR 1—study group; GR 2—control group; ^1^ Fisher’s test; RBC—red blood cells (normal value for females and males: 3.7–5.3 × 10^12^/L); HGB—haemoglobin (normal value: females—7.44–8.57 mmol/L; males: 7.20–8.69 mmol/L); HCT—haematocrit (normal value: females—0.325–0.410 L/L; males: 0.275–0.410 L/L); MCV—mean cell volume (normal value: females—75–87 fL; males: 74–86 fL); MCH—mean corpuscular haemoglobin (normal value: females—1.64–1.80 fmol; males: 1.45–1.86 fmol); MCHC—mean cell haemoglobin concentration (normal value: females—20.50–22.73 mmol/L; males: 20.00–22.73 mmol/L); iron (normal value for females and males: 50–120 µg/dL); transferrin (normal value for females and males: 2.15–3.99 g/L); ferritin (normal value for females and males: 15–120 ng/mL); [32,33]; ns—not significant.

**Table 7:** Dietary intake in children.

Dietary Intake (% RDA)	GR 1 (*n* = 80)	GR 2 (*n* = 80)	*p*
	Median (1st–3rd Quartile)		
Iron ^1^	101.380 (85.630–120.500)	60.200 (43.880–103.510)	<0.001
Proteins ^1^	313.080 (283.780–334.520)	305.910 (265.460–401.880)	ns
Fibre ^1^	130.900 (103.100–179.750)	82.000 (63.900–99.000)	<0.001
Vitamin C ^1^	256.730 (157.920–412.330)	183.600 (116.970–208.880)	<0.001

GR 1—study group; GR 2—control group; RDA—recommended dietary allowance according to Polish nutritional standards [31]; ^1^ Mann–Whitney test; ns—not significant.

## Data Availability

The data presented in this study are available on request from the corresponding author.
